# *Spodoptera frugiperda* Smith fitness on four natural hosts using a two-sex life table in a controlled setting

**DOI:** 10.3389/finsc.2025.1548497

**Published:** 2025-10-01

**Authors:** Alia Tajdar, Chuan Cao, Waqar Jaleel, Syed Muhammad Zaka, Wangpeng Shi

**Affiliations:** ^1^ Department of Entomology, China Agricultural University, Beijing, China; ^2^ Department of Entomology, Faculty of Agricultural Sciences and Technology, Bahauddin Zakariya University, Multan, Pakistan

**Keywords:** Age-stage two-sex life table, castor, fall armyworm, potato, reproduction, *Spodoptera frugiperda*, survival

## Abstract

*Spodoptera frugiperda* (J.E. Smith), (Noctuidae, Lepidoptera), commonly known as fall armyworm (FAW), is a significant polyphagous pest that can cause considerable damage to various crops. Fundamental research on FAW is crucial and beneficial for creating an integrated management strategy. Lot of literatures are available on web to describe the fitness of FAW via conventional methods that deals the basic biology of FAW. However, there is currently a need to check the fitness for each stage of FAW using an advanced two-sex life table tool, which is crucial for creating efficient control strategies. The proposed study used an age-stage, two-sex life table to examine the lifetable parameters of FAW on four natural hosts: castor beans (*Ricinus communis*), potatoes (*Solanum tuberosum*), maize (*Zea mays* L.), and wheat (*Triticum aestivium* L.). The findings demonstrated that, despite notable variations in development and reproduction, the FAW completed its life cycle on each of the four studied hosts. The FAW that were fed maize performed at their best, showing shorter immature (egg-pupa) phases, longer lifespans, and better rates of adult reproduction. On maize, female FAW had the highest fecundity (2497.1 eggs/female), while on wheat, it was the lowest (675 eggs/female). With values of 532.8 (offspring individual-1), 0.21d^-1^, and 1.23 d^-1^, respectively, net reproductive rate, intrinsic rate of increase, and finite rate of increase peaked on maize, while the corresponding parameters were lowest on wheat (94.62 offspring individual^-1^, 0.11 d^-1^, and 1.12 d^-1^, respectively). This study indicates that all host plants can contribute to the development and outbreak of this pest in the absence of its primary host. Therefore, all potential host plants in the area should be thoroughly examined when developing an IPM program against said pest.

## 1 Introduction

Fall armyworm (FAW), *Spodoptera frugiperda* Smith, (Lepidoptera, Noctuidae), has high migratory ability and a wide host range. These attributes collectively play a significant role in causing economic losses to crops and pastures globally (1). The larvae of FAW consume the stems, foliage, and reproductive structures of the plants they inhabit (2). The FAW may prefer or be more effective on one plant species or a small number of host plants (3, 4). Due to its polyphagous nature, FAW can consume over 350 plants from 76 different families, including the Leguminosae, Compositae, and Gramineae (5).

The fall armyworm significantly threatens global food security, causing millions of dollars in losses to maize production areas worldwide (6–8). FAW targets crops from seedlings to maturity, causing physical damage that lowers maize yields. Additionally, it impacts other crops like potatoes, intensifying its economic consequences (9, 10). Farmers have experienced substantial economic losses due to FAW infestations. Prior to its outbreak, maize yields averaged 2–3 tons per acre; however, this dropped to less than 2 tons per acre following its spread (11–13). Native to America, particularly the United States and Argentina, and was initially discovered in central Africa in 2016 (14). It subsequently spread to India (15) and China by 2018; today, it is present across nearly all maize-growing regions of the country (1). In April 2019, for the first time in Sindh, Pakistan, the presence of FAW was confirmed on fodder corn, causing 100% damage to the maize crop (16). The FAW has two biotypes: the corn strain, which primarily invades maize, and the rice strain, which invades rice (17).

In tropical and subtropical areas around the world, FAW populations have developed insecticide resistance as a result of the careless application of pesticides (18), including Asia (7, 19–21). Over the past three decades, interest in behavioral manipulation as a pest management strategy has grown significantly, aiming to reduce dependence on broad-spectrum insecticides (22, 23). Cultivating standing crops is considered a safer and more sustainable alternative to insecticide application (24, 25).

Investigating how insect pests and host plants interact can reveal important details about how host plants affect herbivore biology, ecology, and population dynamics (26–28). Basic research on FAW is essential for creating a trustworthy Integrated Pest Management (IPM) strategy, which includes comprehending its behavior and the parameters of its age-stage, two-sex life table (9, 29, 30). A review article that includes all the components of an integrated pest management for FAW in maize crops was recently published by Babendreier et al. (2022) (31) Traditional life table studies often focus on female age-specific populations (32), but incorporating data on both males and females is essential for a comprehensive understanding of pest dynamics (33–37).

Even though research has been done on the biology of FAW on many hosts (38–42), its host preferences and two-sex life table characteristics are important and necessary to report for best management of FAW. Thus, the purpose of this study was to evaluate, in a laboratory setting, the host preference and age-stage, two-sex life table properties of FAW on the leaves of maize, wheat, castor beans, and potatoes. This study advances our knowledge of FAW and could aid in the creation of more potent control measures.

## 2 Material and method

### 2.1 Laboratory colony

In order to grow them, the FAW larvae were first taken from maize fields in Pakistan’s south Punjab region and taken to the Biological Control Laboratory at the Department of Entomology, Faculty of Agricultural Sciences and Technology, Bahauddin Zakariya University (BZU), Multan. For two or three generations prior to pupation, these larvae had been fed castor leaves. Larvae were raised at a photoperiod of 14:10 hours (L:D), a temperature of 26 ± 1°C, and a relative humidity (RH) of 65 ± 5%. After adult emergence, they were placed in transparent plastic jars (10.16×10.16×17.78cm). Cotton swap soaked in a honey solution (8% w/v) was provided as an artificial diet. Muslin cloth was tapped on two opposite inner sides of the jar as an ovipositional substrate. Each egg mass laid by females was collected and placed separately in a petri dish (2×6 cm) under laboratory-controlled conditions (9).

### 2.2 Plant source

Based on field observations in the various parts of south Punjab where these crops surrounding maize, four host plants were chosen: castor beans (*Ricinus communis*), potatoes (*Solanum tuberosum)*, wheat (*Triticum aestivium* L.), and maize (*Zea mays* L.). The leaves of castor beans and potatoes were gathered from a farm close to Bosan Road in Multan. From the field of BZU Multan, Pakistan, wheat and maize leaves at the V5 stage (the growth point above ground and 1-1½” above the soil surface) were gathered. FAW were fed 30 cm of potato plants, 182.88 cm of caster beans, and the leaves of a V5 stage wheat plant.

### 2.3 Biological parameters

We looked at and contrasted the growth, survival, and reproduction of FAW fed on castor leaves, maize, potatoes, and wheat. Leaves were cut into disc shapes (2×6cm) except wheat leaves, which were cut into 7.62cm, and maize leaves were cut into 2×2cm and replaced with new leaves every 24 hours. Number of leaves varied with the larval instar. From the F3 generation, FAW eggs were collected, and neonates were housed individually in a 2x6 cm petri plate once they hatched. There were 50 replications for every host, and each host was regarded as a separate treatment. The presence of exuvium verified the existence of distinct FAW instars. After adult emergence, the male-female ratio was noted by their morphological characters in each treatment and pairs in different jars (10.16×10.16×17.78cm) to observe oviposition every 24 hours. Cotton swap soaked in honey solution (10% w/v) was provided as an artificial diet and it was changed daily. Up to the female’s death, the egg masses that each female laid were noted every day. After carefully transferring each egg mass to the plastic containers, the number of neonates that hatched was recorded. Fecundity, oviposition period, survival, and female lifespan were assessed.

### 2.4 Statistical analysis

Differences in biological parameters among treatments (different hosts) at each dose were analyzed separately using the Kruskal-Wallis test (P< 0.05). *Post hoc* pairwise comparisons were performed using Dunn’s test in SPSS Statistics 22.0 (SPSS Inc., Chicago, IL, USA). These non-parametric tests were employed after confirming through normality test that the data not followed the normal distribution. Using a TWO SEX-MS Chart, the life table parameters of FAW individuals were determined, age stage specific *f_xj_*: fecundity, *l_x_*: survival rate, *s_xj_*: specific survival rate, *l_x_m_x_*: maternity, *m_x_*: specific fecundity, *e_xj_*: life expectancy, *v_xj_*: reproductive value, and population parameters, Net reproductive rate (*R*_0_ *)*, intrinsic rate of increase (*r*), finite rate of increase (*λ*), and mean generation time (*T*) (43) were calculated as (1 and 2):


(1) $$l_{x}=\sum_{j=1}^{k}S_{xj}$$

(2) $$m_{x}=\frac{\sum_{j=1}^{k}S_{xj}f_{xj}}{\sum_{j=1}^{k}S_{x}}$$

(3) $$R_{0}=\sum_{x=0}^{\infty }l_{x}m_{x}$$

by following the Euler–Lotka Equation 4, with age indexed from (44):


(4) $$\sum_{x=0}^{\infty }e^{-r\left(x+1\right)}l_{x}m_{x}=1$$

(5) $$\lambda =e^{r}$$

(6) $$T=In\;R_{0}/r$$

Life expectancy (exj) using the formula provided in Equation 7 (45).


(7) $$e_{xj}=\sum_{i=x}^{\infty }\sum_{y=j}^{\beta }S{/}_{iy}$$

The Vxj was calculated by using the following equation (46).


(8) $$V_{xj}=\;\frac{e^{-r\left(x+1\right)}}{S_{xj}}\sum_{i=x}^{\infty }e^{-r\left(x+1\right)}\sum_{y=j}^{k}S_{iy}f_{iy}$$

## 3 Results

### 3.1 Life span of FAW

When fed on various host plants, the female reproductive capacity (FAW), adult lifespan, and development length for each immature stage differed considerably according to the Kruskal-Walli’s test and Dunn’s test at *p* < 0.05 (**Table 1**). Each egg stage lasted roughly two days since freshly hatched neonates were consistently recovered from egg masses. On maize and potatoes, every immature stage from the first instars to the pupa developed noticeably more quickly (all *p <* 0.05). When given different host plants as food, males (H = 42.158; *df* = 3, *p* < 0.0001) and females (H = 40.722; *df* = 3, *p <* 0.001) showed significantly varying adult longevity. When fed maize, the male and female lived the longest (18.14 and 18.57 days, respectively), whereas when fed wheat, they lived the shortest (9.43 and 10.86 days, respectively). Significant variations (H = 29.278; *df* = 3, and *p* < 0.0001) in the pre-oviposition period were noted when the FAW were fed on various plants (**Table 1**). Additionally, when FAW was fed on several host plants, the oviposition duration varied significantly (H = 37.473; *df* = 3, and *p* < 0.0001). On maize, the oviposition period peaked at 6.7 days, although other hosts displayed a comparable pattern. On several host plants, the fecundity of FAW varied considerably (H = 33.871; *df =3*, and *p* < 0.0001); Maize had the highest fecundity (2497.1 eggs/female), followed by castor (2194.6 eggs/female); the statistic ranks of potatoes (1166 eggs/female) and wheat (675 eggs/female) were comparable.

**Table 1:** Fall armyworm growth and reproductive characteristics (mean ± SE) on several host plants.

Developmental stages	Hosts								Statistical parameters		
	Castor	N	Potato	N	Wheat	N	Maize	N	H (statistics)	*df*	*p-*value
Egg	2.00a ± 0.00	49	2.00a ± 0.00	40	2.00a ± 0.00	46	2.00a ± 0.00	50			
1st instar (d)	2.46b ± 0.20	46	4.86a ± 0.38	40	4.44a ± 0.27	46	1.38c ± 0.10	49	139.300	3	<.0001
2nd instar (d)	2.12a ± 0.17	46	1.24b ± 0.14	36	2.76a ± 0.23	45	1.52b ± 0.12	46	77.380	3	<.0001
3rd instar (d)	1.60b ± 0.11	40	1.14c ± 0.12	36	2.30a ± 0.20	42	1.46bc ± 0.13	43	50.629	3	<.0001
4th instar (d)	1.94a ± 0.15	40	1.42b ± 0.14	35	1.80a ± 0.16	42	1.94a ± 0.17	41	26.028	3	<.0001
5th instar (d)	1.76b ± 0.14	40	2.12b ± 0.22	35	3.00a ± 0.25	41	1.76b ± 0.18	40	55.821	3	<.0001
6th instar (d)	2.92a ± 0.24	40	2.90a ± 0.32	32	3.44a ± 0.29	37	3.36a ± 0.30	38	10.221	3	0.0153
Pupa (d)	7.96a ± 0.68	30	4.80b ± 0.69	24	3.60c ± 0.70	18	4.76b ± 0.51	33	77.806	3	<.0001
Whole span from egg to pupae (d)	22.76a ± 1.53	30	18.48b ± 1.63	24	23.34a ± 1.36	18	18.18b ± 1.05	33	74.674	3	<.0001
Male longevity (d)	13.29b ± 0.71	15	12.00b ± 1.40	10	9.43b ± 1.59	7	18.14a ± 0.99	16	42.158	3	<.0001
Female life (d)	16.00ab ± 2.49	15	14.71bc ± 1.57	14	10.86c ± 0.74	11	18.57a ± 0.69	17	40.722	3	<.0001
Preoviposition (d)	7.57a ± 0.75	15	5.00b ± 0.72	14	5.43b ± 0.57	11	5.00b ± 0.38	16	29.278	3	<.0001
Oviposition (d)	4.60b ± 0.48	15	4.1b ± 0.34	14	3.40b ± 0.78	11	6.7a ± 0.61	16	37.473	3	<.0001
Postoviposition (d)	3.86bc ± 0.59	15	5.71ab ± 1.35	14	2.43c ± 0.75	11	5.86a ± 0.261	16	40.079	3	<.0001
Fecundity (eggs/female)	2194.6a ± 292.34	15	1166.7b ± 308.69	14	675.00b ± 210.8	11	2497.1a ± 283.57	16	33.871	3	<.0001

Data are presented as mean ± SE. Different lowercase letters within a row indicate significant differences (*P* < 0.05) based on the Kruskal-Wallis one-way ANOVA, followed by Dunn’s test for pairwise comparisons. df, degrees of freedom; H: Statistics Value.

### 3.2 Population parameters

The intrinsic rate of increase (*r*), finite rate of increase (*λ*), the net reproductive rate (*R_0_*), and mean generation time (*T*) of FAW on different hosts were assessed using the bootstrap method, and are listed in **Table 2**. Statistical analysis showed that intrinsic rate of increase (*r)*, finite rate of increase (*λ*), and net reproductive rate (*R_0_*) were higher on maize provided as food than with the other tested plants (**Table 2**). The mean generation time *T* was higher (39.02 d^-1^) on wheat, followed by castor and potato, while shorter on maize. When fed castor leaves, the FAW produced offspring with a Gross reproductive rate (*GRR*) of 973.17, followed by offspring fed maize, potatoes, and wheat with *GRR*s of 833.70, 408.66, and 406.11, respectively.

**Table 2:** The population parameters of Fall armyworm fed on 4 different hosts.

Parameters	Castor	Potato	Wheat	Maize
Intrinsic rate of increase, *r* (d^−1^)	0.16	0.14	0.11	0.21
Finite rate of increase*, λ* (d^−1^)	1.17	1.15	1.12	1.23
Net reproductive rate, *R_0_*(offspring)	424.36	174.94	94.62	532.8
Mean generation time, *T* (d)	36.95	35.17	39.02	30.0
The gross reproductive rate (GRR)	973.17	406.11	408.66	833.70

### 3.3 Survival rate

**Figure 1:**
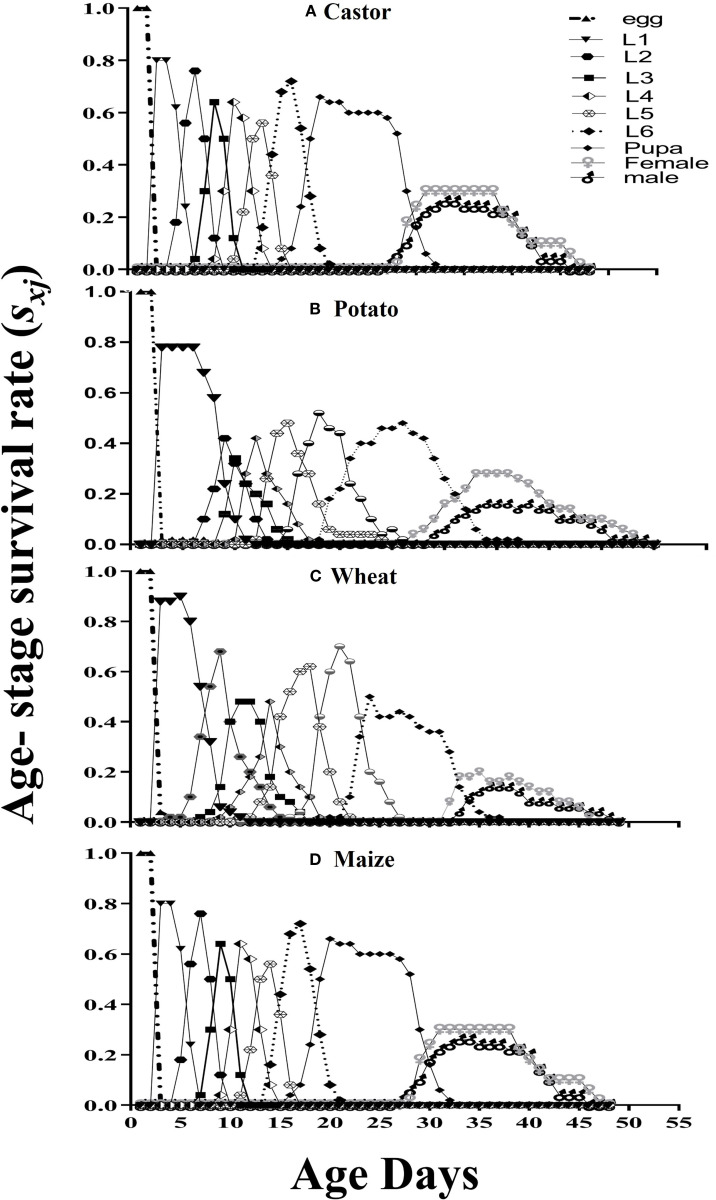
Age-stage survival rate (*Sxj*) for fall armyworm fed on four different host plants: **(A)** castor, **(B)** potato, **(C)** wheat, and **(D)** maize, under laboratory conditions.

When fed maize, the greatest survival probability survival rate (*s_xj_*) of FAW pupa and eggs was 0.86 and 1, respectively. The survival probability of all larval stages was highest when they were nourished on Castor leaves. The maximum survival probability of males and females were 0.421 and 0.447, respectively on Maize leaves, while the least survival probability (0.189 and 0.290) was observed on wheat (**Figure 1**).

### 3.4 Population survival rate and fecundity

**Figure 2:**
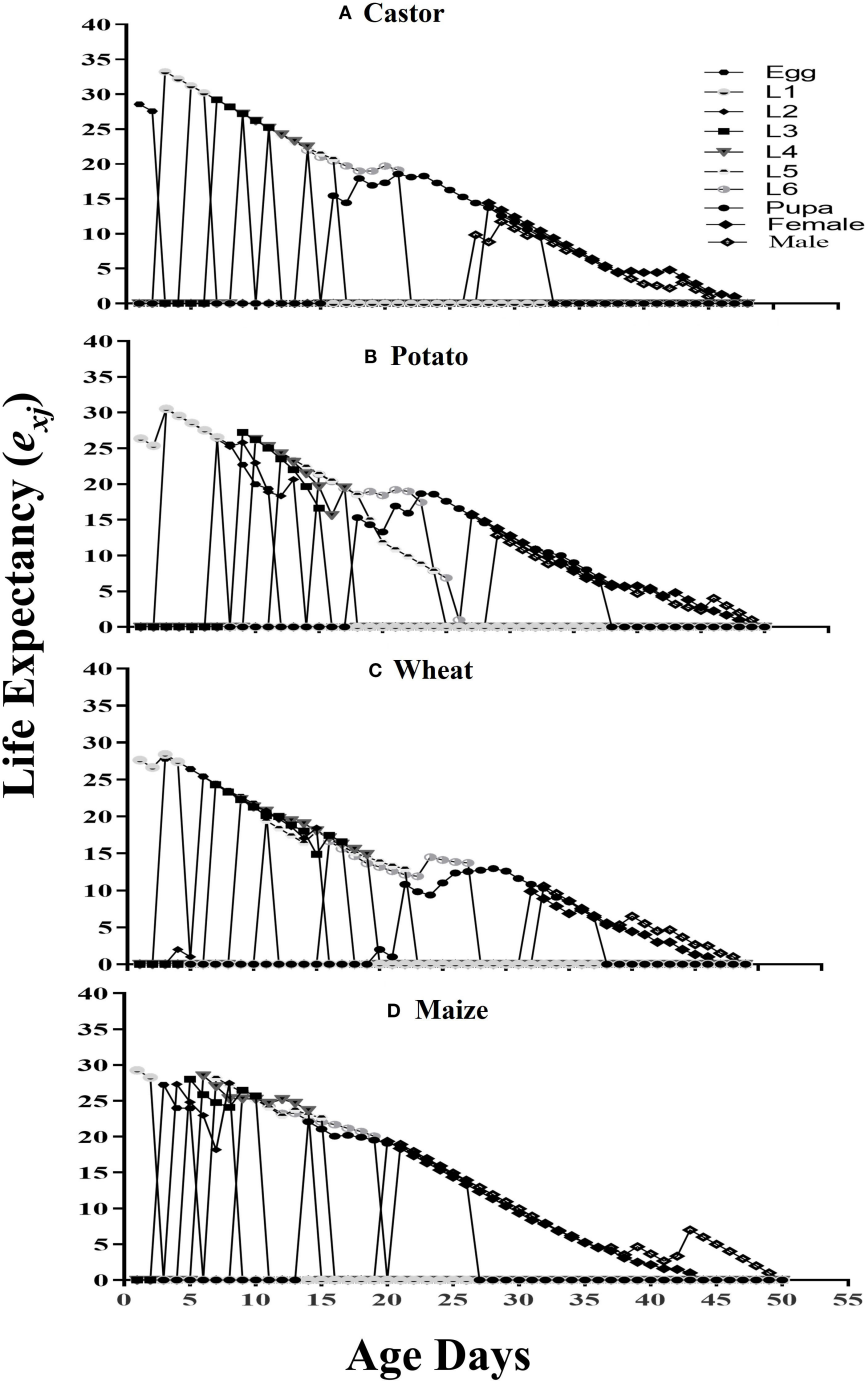
The survival rate (*l_x_*), the fecundity (*f_xj_*), the specific fecundity (*m_x_*) and the maternity (*l_x_m_x_*) of fall armyworm fed on four different host plants: **(A)** castor, **(B)** potato, **(C)** wheat, and **(D)** maize, under laboratory conditions.

**Figure 3:**
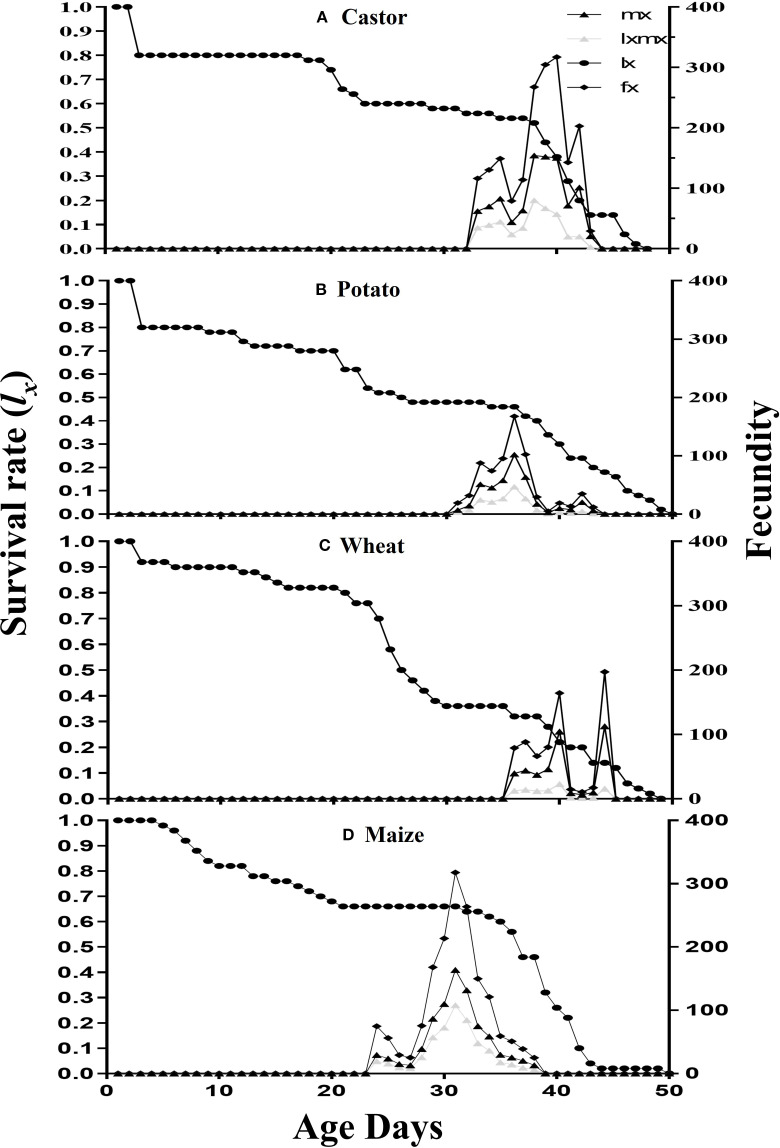
The reproductive value (*V_xj_*) of fall armyworm fed on different host plants: **(A)** castor, **(B)** potato, **(C)** wheat, and **(D)** maize under laboratory conditions.

The survival rate (*l_x_*), fecundity (*f_x_*), age-specific fecundity (*m_x_*), and age-specific maternity rate (*l_x_m_x_*) are plotted in **Figure 2**. The survival rate *l_x_* has shown maximum on both maize and castor than on wheat and potato. The fecundity (*f_x_)* showed that 317.22eggs on the 39^th^ d, 167.64eggs on the 35^th^d, 197.25eggs on the 43^th^d, and 317.70eggs on the 30^th^day were laid on castor, potato wheat, and maize, respectively. The age-specific fecundity (*m_x_*) curve showed that reproduction began at 29d, 34d, 37d, and 38d in FAW fed on maize, potato, castor, and wheat, respectively. The age-specific maternity rate (*l_x_m_x_*) of FAW was maximum on maize followed by castor, potato, and wheat. The values of the reproductive value (*v_xj_*) of an adult female were recorded with the following trend: 813.61at the 28^th^d on maize, 739.89 at the 32d on castor, 408.44 on the 43^rd^ day on potato, and 386.84 at the 55^th^d on wheat (**Figure 3**).

### 3.5 Life expectancy

**Figure 4:**
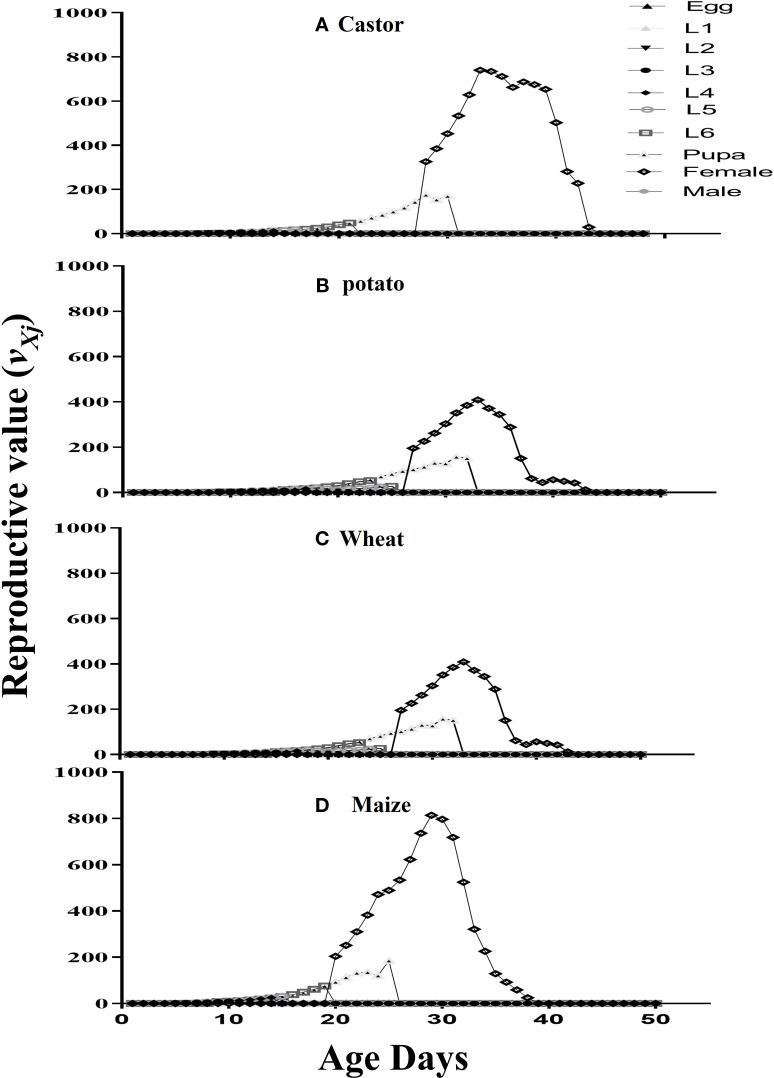
The life expectancy (*e_xj_*) of fall armyworm fed on different host plants: **(A)** castor, **(B)** potato, **(C)** wheat, and **(D)** maize, under laboratory conditions.

The estimated duration of survival for an insect of age x and stage j is denoted by the life expectancy (*exj*) (**Figure 4**). Freshly deposited FAW eggs raised on maize, castor, potato, and wheat had respective life expectancies of 29.26, 33.2, 30.53, and 28.40.

## 4 Discussion

The biology of herbivorous insects, including FAW is significantly impacted by variations in the nutrient content of their host plants and also affects the shifting trends of their populations (9, 47–54). A plant is deemed more suitable when an insect feeds on it and shows signs of faster development and higher reproduction rates (55). Our findings showed that FAW completed its life cycle on all hosts, and the total duration of immature development (from egg to pupa) was shorter on maize than on other tested hosts. Additionally, adult longevity (both male and female) and fecundity were higher on maize than on other treatments. Xu et al.,(2019) observed that when FAW larvae were fed tobacco (a non-preferred host) instead of maize, their development time was extended, while survival rate and fecundity were decreased, which aligns with our findings (56). Likewise, Wu et al. (2020) reported that FAW exhibited faster development and heavier pupal weight and fecundity when reared on primary hosts such as wheat and maize (1). Similarly, another study revealed that FAW could complete its life cycle on both hosts (kidney beans and maize). The larval and pupal development duration was notably extended, while the adult lifespan was shortened on kidney beans compared to maize, with no difference in the oviposition rate (57). According to Acharya et al. (2022), maize had higher reproduction rates and a shorter mean generation time than rice and potatoes (58). Various factors can cause these differences among host plants, including extrinsic factors such as food source characteristics and intrinsic genetic characteristics (59–61). The reduced performance observed in other test plants may be linked to insufficient nutrition and the presence of certain insect-repelling compounds.

The life parameter statistics the intrinsic rate of increase (*r*), the finite rate of increase (*λ*), the net reproductive rate (*R_o_*), and the mean generation time (*T*) offer valuable insights into the growth potential of a population in a specific environment (62). These parameters frequently change depending on the type of host plant and the environmental conditions of the area (42, 57, 63). Our findings showed that shorter developmental time, high oviposition period, and high fecundity rate of FAW reared on maize resulted in higher the intrinsic rate of increase (*r*), the finite rate of increase (*λ*), the net reproductive rate (*R_0_*), and lower the mean generation time (*T*) values. The findings are consistent with the previous study, which demonstrated that when FAW reared on maize, it exhibits a higher capacity for population growth (higher rates of *r*, l, *R_0_*, and a lower *T*) compared to tomato, cotton, or soybean (1). Similar findings on maize were reported by Wu et al. (62), with the exception that tomato had the highest net reproductive rate. Likewise, higher intrinsic rate of increase (*r*), the finite rate of increase (*λ*), the net reproductive rate (*R_0_*), and lower *T* values were recorded previously when FAW was raised on maize compared to potato and rice (58). A similar trend was observed when FAW was raised on maize instead of kidney beans (57). These trends may be attributed to the nutritional differences found in the host plants (64).

The survival probability of all larval instars was higher on wheat; however, the maximum survival rate of males and females was observed on maize in comparison with other hosts. The life expectance (*e_xj_*) also varied among the four host plants. The life expectance (*e_xj_*) of freshly laid eggs of FAW was greater on castor (33.2), followed by potato (30.53), maize (29.26), and wheat (28.40). Following our results, a previous study reported that the survival rates of newly hatched FAW neonates to adult age showed considerable variation across three host plants: maximum survival was observed on maize (98.31%), followed by potato (31.61%) and tobacco (8.13%) (9). The differences in results may be due to variations in laboratory conditions (temperature, humidity, and light), as well as sample preparation, handling, and measurement techniques. Moreover, genetic variations among maize cultivars or different strains of organisms could also influence the outcomes. Likewise, the survival rate and life expectancy of FAW were greater in maize compared to kidney beans (57). According to Altaf et al. (2022) reported that the Survival rate and life expectancy of FAW were higher in maize than in sorghum, wheat, and rice under laboratory conditions (65). These parameters are used to develop early warning models that predict the survival of insects at a certain age, timing, and amount of pest occurrence (28, 66).

To accomplish dependable Integrated Pest Management (IPM) (31), basic research of FAW are required, including behavior and age-stage, two-sex life table factors (9, 30). Traditional life table characteristics, which only offer data for female age-specific populations, are frequently used to study the development and survival of pests (32). Tobetter comprehend a pest, life table parameters for both males and females are required (33, 34, 36). Although the biology of FAW on several hosts has been researched (9, 38, 67), there is a dearth of information on your host and the two-sex life table characteristics of FAW. Furthermore, research on FAW’s preferred food has shown that growth, survival, and effectiveness are contingent upon the availability of food sources and favorable environmental conditions. To the best of our knowledge, however, no thorough data utilizing age-stage, two-sex life methods has previously been published on the fitness of the FAW on host (9, 49). Fall Armyworm (FAW) in maize is controlled via Integrated Pest Management (IPM), which takes a multifaceted strategy to reduce the pest’s negative effects on the environment (31, 68, 69). In order to interrupt the FAW life cycle, cultural methods like crop rotation, intercropping, and optimal planting schedules are essential, as are routine monitoring for early diagnosis and prompt interventions (70–73). Natural predators, parasitoids, entomopathogenic fungi and plant extracts are examples of biological control techniques that can naturally lower FAW populations and are more environmentally benign than chemical control (74–76). Since two-sex life table studies take into consideration both sexes when creating the appropriate population curve for future populations, they aid researchers in creating pest management plans to combat any pest (43, 45, 77, 78).This helps us comprehend the ecology and fitness of an insect pest and improve its management, as it was reported by Zafar et al. (2024) (79). The reproductive characteristics *r*, GRR *λ*, and *R_0_*values are important in determining how diet impacts the fitness of insect pests (34). If *r* is greater than 0, it might be the best population index to show how an insect has adapted to a food source (80, 81). Our research underscores the substantial impact of host plant variability on FAW’s life cycle and population dynamics. Understanding these dynamics can inform pest management strategies and contribute to more effective control measures by considering the host plant’s influence on insect fitness and population growth. Future research can involve the construction of life tables in field conditions to better understand the ecology of the FAW population in Asian maize cropping environments.

## 5 Conclusions

The life table parameters of FAW were studied on different hosts i.e., maize, castor, potato, and wheat, via the age-stage two-sex method. Our findings revealed that the FAW was able to complete its life span on all four host plants; however, it exhibited a shorter pre-adult duration, a higher survival rate, and greater fecundity on maize when compared to castor, potato, and wheat. The interaction of all these parameters resulted in increased the net reproductive rate (*R_0_*), the intrinsic rate of increase (*r*), and the finite rate of increase (*λ*) in the FAW raised on maize, highlighting the pest’s strong adaptability to maize compared to other tested host plants. FAW performed much better in maize (primary host), the establishment of an integrated pest management program for FAW in maize is highly recommended to suppress its population and minimize economic losses. Furthermore, our results propose that castor, potato, and wheat can serve as alternative hosts for FAW, allowing it to complete its life cycle even when preferred crops are absent. Consequently, it is essential to monitor alternative host plants to observe their population changes and assess any potential crop damage.

## Data Availability

The original contributions presented in the study are included in the article/supplementary material. Further inquiries can be directed to the corresponding authors.
